# A Novel Prediction Method about Single Components of Analog Circuits Based on Complex Field Modeling

**DOI:** 10.1155/2014/530942

**Published:** 2014-07-22

**Authors:** Jingyu Zhou, Shulin Tian, Chenglin Yang

**Affiliations:** School of Automation Engineering, University of Electronic Science and Technology of China, Chengdu 611731, China

## Abstract

Few researches pay attention to prediction about analog circuits. The few methods lack the correlation with circuit analysis during extracting and calculating features so that FI (fault indicator) calculation often lack rationality, thus affecting prognostic performance. To solve the above problem, this paper proposes a novel prediction method about single components of analog circuits based on complex field modeling. Aiming at the feature that faults of single components hold the largest number in analog circuits, the method starts with circuit structure, analyzes transfer function of circuits, and implements complex field modeling. Then, by an established parameter scanning model related to complex field, it analyzes the relationship between parameter variation and degeneration of single components in the model in order to obtain a more reasonable FI feature set via calculation. According to the obtained FI feature set, it establishes a novel model about degeneration trend of analog circuits' single components. At last, it uses particle filter (PF) to update parameters for the model and predicts remaining useful performance (RUP) of analog circuits' single components. Since calculation about the FI feature set is more reasonable, accuracy of prediction is improved to some extent. Finally, the foregoing conclusions are verified by experiments.

## 1. Introduction

Prognostics and health management (PHM) is very important to the reliability study on one product life cycle. PHM includes two aspects, namely, diagnosis and prognostics. Research on fault diagnosis to analog circuits has rapid growth [1, 2]. For example, many methods have been put forward in researches on fault model, tolerance, and nonlinearity [3–8]. In early stage, Pan and Cheng propose an economical and effective parametric fault diagnosis method to linear time invariant circuits [6]. In this method, perceptron is used to obtain test sequence which is used as input for fault diagnosis and fault classification of device under test (DUT). Long et al. propose a test generation algorithm to analog circuits based on support vector machine (SVM) [7]. In this algorithm, SVM is used to map nonlinear response space onto high-dimensional space so as to conduct effective fault classification. Long et al. propose a tolerance-considered fault diagnosis method to analog filter circuits [8]. This method is based on least square support vector machine (LS-SVM) and treats frequency output as fault feature.

Because fault diagnosis to analog circuits always takes effect after the fault generates, fault diagnosis cannot effectively prevent failure. Therefore, compared to fault diagnosis, prognostics study plays a more positive and active role in preventing system failure. Major prognostics include prognostics based on physics of failure (PoF) and data-driven prognostics. Prognostics based on physics of failure rely on features obtained from measurement, such as voltage, current, and temperature. PoF cannot be easily used to fault prognostics to analog circuits due to tolerance and features continuity. Therefore, to prediction method of analog circuits, data-driven prediction method can be accepted more easily in practical application.

Although many methods of data-driven prediction have been put forward, they are rarely used in prediction on analog circuits. Vasan et al. put forward a prediction method about analog circuits [9]. In this method, fault indicator (FI) is calculated through Mahalanobis distance. Because Mahalanobis distance reflects small change of features in an excessively obvious way, influence of introduced parameter tolerance is enlarged and front features of degradation model have big fluctuation, which greatly reduces integrity of prognostics information and accuracy of prognostics. Li et al. put forward a prediction method about analog filter circuits based on particle filter (PF) through using frequency features [10]. In this method, fault indicator is calculated through several special parametric features extracted from frequency output signals to predict remaining useful performance (RUP). This prediction method neglects relevance between two different features, making FI calculation less reasonable.

In order to solve the foregoing problems, this paper mainly aims at prediction on single components of analog circuits, begins with analyses of circuit structure, and proposes a prediction method about single components of analog circuits based on complex field modeling to predict RUP of analog circuits' single component. The difference between the method and previous ones is shown as follows. Firstly, the method carries out complex field modeling by analyzing transfer function of circuits and analyzes the relationship between parameter variation and degeneration of single components in the model by an established parameter scanning model related to complex field. In doing so, more reasonable FI is obtained by calculation. Then, according to newly obtained FI, it establishes a novel model about degeneration trend of analog circuits' single components. To compare with previous algorithms, particle filter is applied to adapt to the model and predict RUP of analog circuits' single components.

In the following content, this paper will be divided into several parts to expound the prediction method.  Section 2 gives an overview about structure of the prediction method proposed by this paper; Section 3 states FI calculation and collection of prediction on single components of analog circuits based on complex field modeling; Section 4 explains the degradation model and the prediction method based on the degradation model in this paper; Section 5 elaborates steps and procedures of the prediction method in this paper; Section 6 puts forward performance of the prediction method put forward in this paper via simulation and experimental verification; and Section 7 draws a conclusion.

## 2. General Introduction to Overall Framework of Prediction on Analog Circuits Proposed in This Paper

**Figure 1:**
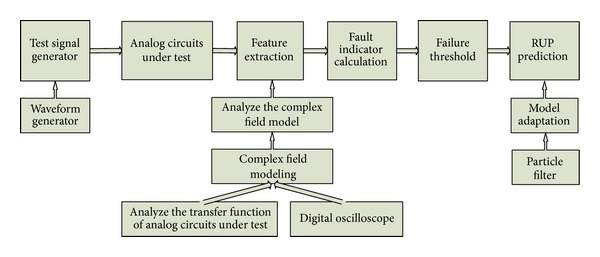
Framework of prediction on analog circuits proposed in this paper.

Framework of prediction on analog circuits proposed in this paper is as shown in Figure 1.

From circuit performance, it can be seen that with time change, negative influence of components degradation on circuit performance is always nonlinear. However, in consideration of long components life cycle (e.g., a general resistance has a lifetime of up to 5–10 years), we may assume that change tendency of fault level is on increase along with the time and that fault level increases by a small constant under time index (in [9, 10], fault level is assumed to increase by 0.4% along with the time index). According to above analysis, continuous degradation process of a circuit can be considered as the sum of many discrete steps. In other words, in order to discuss performance prognostics to analog circuit, it would be well if increase value of components fault level is set at a small constant and the other components values are set within a tolerance range. In addition, the number of prognostic components only can be set at one in this paper.

For prediction on analog circuits, all previous thoughts extracted some features from predicted circuits and obtained FI by arithmetical operation according to extracted features to establish the degradation model. As shown in Figure 1, to combine structure of analog circuits with prediction, the method analyzes the transfer function of predicted circuits first. When parameter scanning of components is implemented, it is found that voltage output of predicted analog circuits basically satisfies a round track model in the complex field. In some cases, the round model will degrade into a linear one. Thus, the method introduces the principle that three points that do not stay in a line may construct a circle and three points that stay in a line may confirm a straight line, carries out round modeling or linear modeling of the complex field by testing output voltage of the three states (fault-free point, open-circuit point, and short-circuit point) and then analyzes the mode. In this way, it judges the relationship between the component degeneration under each time index and parameter variation of the model. In another word, under the round model, it uses the relationship between changes in a central angle and component variation under each time index to establish a FI degradation model; under the linear model, it utilizes the relationship between changes in Euclidean distance and component variation under each time index to build the FI degradation model. Finally, it finishes prediction on RUP of analog circuits by using the FI degradation model proposed in this paper and particle filter to finish update of model parameters under some failure threshold values.

## 3. FI Calculation and Collection of Prediction on Single Components of Analog Circuits Based on Complex Field Modeling

In analyses of measurability, complex-field round modeling theory has been studied to some extent [11]. However, it has not been studied in prediction on analog circuits. This paper puts forward a prediction method about analogy circuits based on complex field modeling theory.

### 3.1. Complex-Field Round Modeling Theory about Analog Circuits

**Figure 2:**
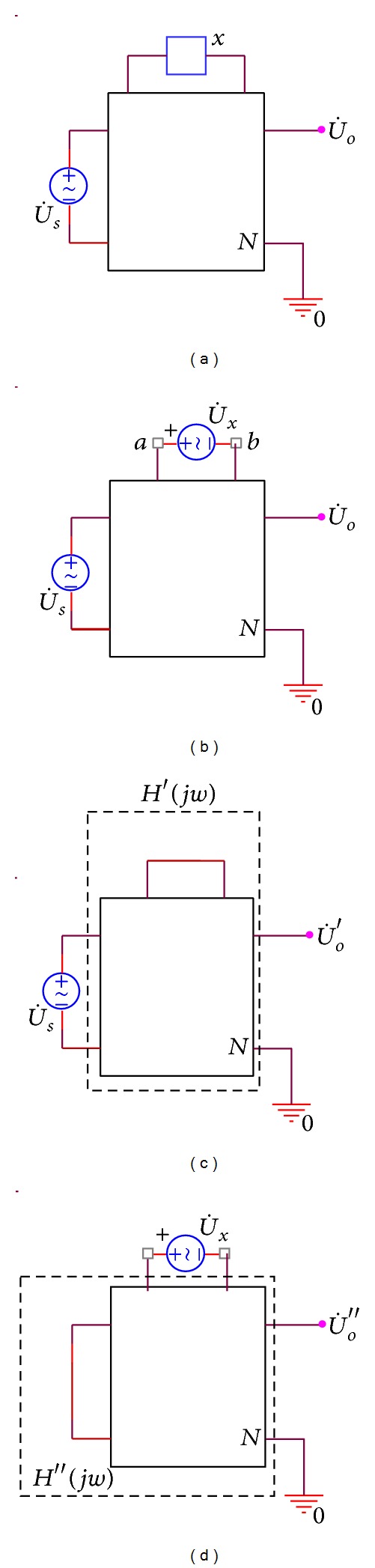
(a) Nominal network, (b) equivalent network, (c) *N* stimulated by *U*_*s*_, (d) *N* stimulated by *U*_*x*_.

As shown in Figure 2(a), *N* represents linear time invariant circuit, voltage phasor of its independent excitation voltage is ${\dot{U}}_{s}$ , voltage phasor of output is ${\dot{U}}_{o}$ , and *x* is a passive component. According to substitution theorem, Figure 2(a) may be converted into Figure 2(b). As shown in Figure 2(b), ${\dot{U}}_{x}$ is the voltage phase passing *x*. Then, according to Thevenin's theorem, (1) can be obtained as follows:

(1) $$\begin{array}{c} {\dot{U}}_{x}={\dot{U}}_{\text{oc}}\frac{Z_{x}}{Z_{0}+Z_{x}}, \end{array}$$

where ${\dot{U}}_{\text{oc}}$ is the open-circuit voltage phasor between *a* and *b* ports in Figure 2(b), *Z*_0_ is a resistance value between *a* and *b*, and *Z*_*x*_ is a resistance value of *x*. In accordance with Thevenin's theorem, ${\dot{U}}_{o}$ in Figure 2(a) is equal to ${\dot{U}}_{o}$ in Figure 2(b). Next, according to superposition principle, Figures 2(c) and 2(d) can be obtained. Thus, (2) can be achieved as follows:

(2) $$\begin{array}{c} {\dot{U}}_{o}^{\prime }={\dot{U}}_{s}H^{\prime }\left(j\omega \right),\\ {\dot{U}}_{o}^{\prime \prime }={\dot{U}}_{x}H^{\prime \prime }\left(j\omega \right), \end{array}$$

where *H*′(*jω*) and *H*′′(*jω*) are the transfer function when ${\dot{U}}_{s}$ and ${\dot{U}}_{x}$ have effect alone, and they are independent of *x*.

Thus, according to the superposition principle, we can get

(3) $$\begin{array}{c} {\dot{U}}_{o}=\;{\dot{U}}_{o}^{\prime }+{\dot{U}}_{o}^{\prime \prime }={\dot{U}}_{s}H^{\prime }\left(j\omega \right)+{\dot{U}}_{x}H^{\prime \prime }\left(j\omega \right). \end{array}$$

By substituting it into (1) and eliminating ${\dot{U}}_{x}$ , we can obtain

(4) $$\begin{array}{c} \frac{Z_{0}}{Z_{x}}=\frac{{\dot{U}}_{\text{oc}}H^{\prime \prime }\left(j\omega \right)}{{\dot{U}}_{os}}-1, \end{array}$$

where

(5) $$\begin{array}{c} {\dot{U}}_{os}={\dot{U}}_{0}-{\dot{U}}_{s}H^{\prime }\left(j\omega \right). \end{array}$$

Without loss of generality, order

(6) $$\begin{array}{c} Z_{0}=R_{0}+jX_{0},\\ {\dot{U}}_{os}=U_{or}+jU_{oj},\\ {\dot{U}}_{\text{oc}}H^{\prime \prime }\left(j\omega \right)=m+jn. \end{array}$$

By substituting (6) into (1), we can achieve

(7) $$\begin{array}{c} \frac{R_{0}+jX_{0}}{Z_{x}}=\frac{mU_{or}+nU_{oj}}{U_{or}^{2}+U_{oj}^{2}}-1+j\frac{nU_{or}-mU_{oj}}{U_{or}^{2}+U_{oj}^{2}}. \end{array}$$

Here, *Z*_*x*_ = *R*_*x*_ + *jX*_*x*_. Since this paper pays attention to prediction on single components, the component *x* only needs to consider a single resistance or capacitance when modeling is implemented; that is, *Z*_*x*_ = *R*_*x*_ or *Z*_*x*_ = *jX*_*x*_. Regardless of the situation that the component *x* is resistance or capacitance, the following equation can be obtained by eliminating *Z*_*x*_ when it is a single component:

(8) $$\begin{array}{c} {\left(U_{or}-c_{r}\right)}^{2}+{\left(U_{oj}-c_{j}\right)}^{2}=r^{2}. \end{array}$$

According to the foregoing equation, it is known that the output voltage (*U*_*or*_, *U*_*oj*_) must satisfy (8) when *x* changes. However, as (8) is an arc whose center is (*c*_*r*_, *c*_*j*_) and radius is *r*, the parameters *c*_*r*_, *c*_*j*_, *r* are composed of other parameters that are independent of *x*. Therefore, it is known that the output voltage must satisfy the arc equation (8) no matter how *x* changes. In this way, complex-field modeling about the round model is realized in the aspect of mathematical model.

### 3.2. FI Collection and Calculation Based on Complex-Field Modeling about the Round Model

**Figure 3:**
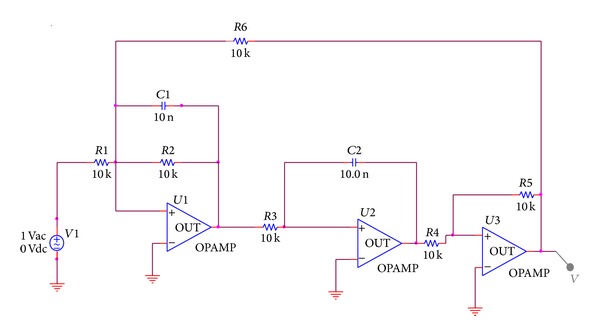
Two-Thomas filter circuit.

**Figure 4:**
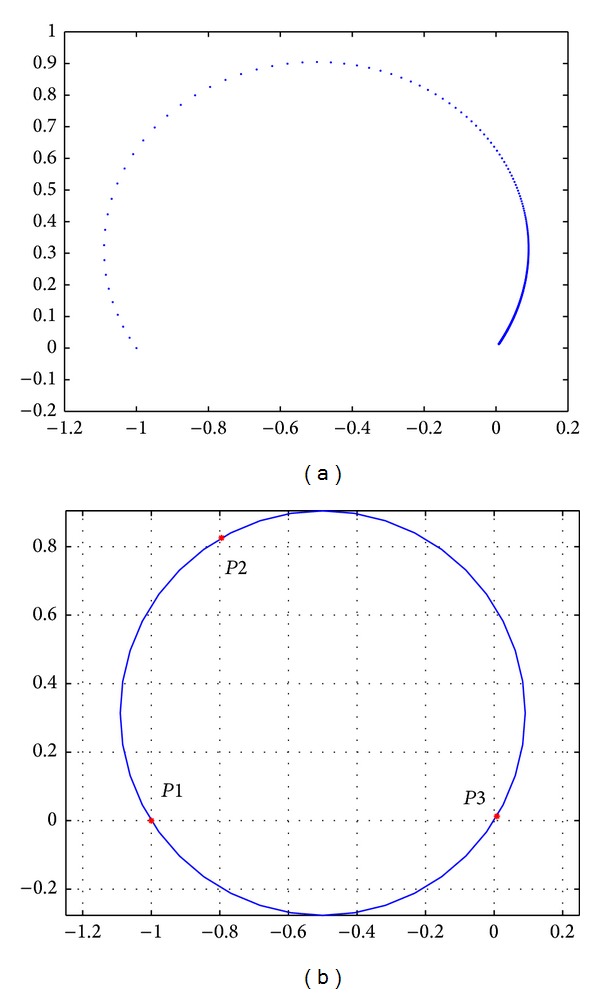
(a) The voltage round model of *R*3 component scanning, (b) the round model obtained by *R*3 three-point circle construction.

In accordance with the theory related to complex-field modeling about the round model in the foregoing section, take Figure 3 Two-Thomas filter circuit as an example. By scanning output voltage of the component *R*3 changing from short circuit to open circuit, Figure 4 can be obtained, where open circuit of component value is set as *R*3 × 10^4^, short circuit is *R*3 × 10^−4^, and scanning step is 0.1 kΩ. Voltage arc of component scanning is shown in Figure 4.

As shown in Figure 4(a), it is found that a component's change from open circuit to short circuit satisfies an arc model. However, for prediction, we cannot obtain a whole arc by the method of component scanning. In addition, the way to infer center and radius of an arc by the transfer function is too complicated. Thus, with regard to establishment of the round model in the process of prediction, this paper proposes that round modeling is carried out by using the output point when the predicted component stays in open circuit, in short circuit, and without faults according to the theory that three points which do not stay in a line may construct a circle. As shown in Figure 4(b), the three points *P*1, *P*2, and *P*3 represent output voltage of the circuit when the component encounters open circuit, short circuit, and no faults, respectively. Via the three points, round modeling is performed well.

As shown in Figure 4(a), for component parameter scanning, points on its arc satisfy a round track all the time. Nevertheless, they are not distributed with equal step on the arc. Thus, after the arc has been built, tracks of the circle are not so important. What is really important lies in parameters affecting distribution of points on the arc when the component changes. In other words, it is assumed that when component degrades at a constant speed according to some time indexes, output does not run at a constant speed although it runs on the circle's tracks. Central angle reflects the process of this nonuniform motion well. For a circle, its radius and location of its center can be determined by the round model that the transfer function deduces. Besides, calculation of the transfer function is too complicated. Therefore, in accordance with analyses of Figure 4(b), this paper proposes that the way to construct a circle by three points can be used to determine related parameters of the circle, for instance, radius. Then, according to changes in related parameters and tracks, calculate the central angel and use it as FI of prediction.

Based on the foregoing analyses, obtain the radius of the round model by the way to construct a circle by three points first. Next, calculate FI of prediction as follows:

(9) $$\begin{array}{c} {\text{FI}}_{t}=\theta_{\Delta t}=2\arcsin\frac{\sqrt{{\left(x_{t}-x_{0}\right)}^{2}+{\left(y_{t}-y_{0}\right)}^{2}}}{2r}. \end{array}$$

When a certain component degrades by *t* time indexes, FI value of prediction is FI_*t*_ and output voltage is *U*_*t*_ = *x*_*t*_ + *jy*_*t*_. Besides, fault-free and displacement-free output is *U*_0_ = *x*_0_ + *jy*_0_ and *r* is the radius of the round model related to circle construction by three points.

**Figure 5:**
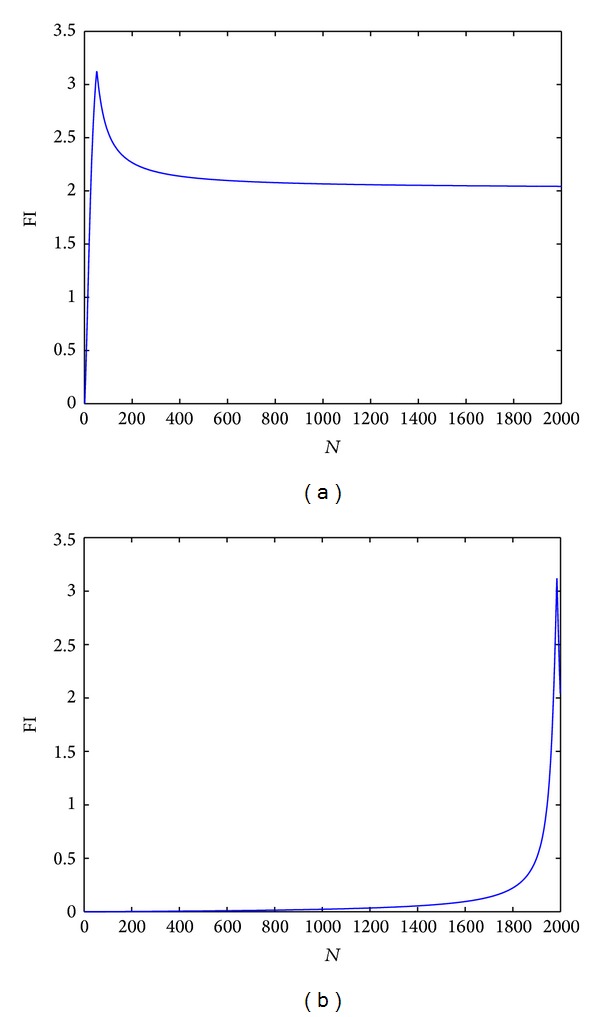
(a) The scanning of the component *R*3 from short circuit to open circuit, (b) FI about the scanning of the component *R*3 from open circuit to short circuit.

According to (9), obtaining a FI changing curve by scanning a component from short circuit to open circuit without loss of generality is in order to analyze the feasibility and generalization corresponding to the method of FI calculation. Take component *R*3 as an example, carry out the scanning whose step is 5 kΩ from open circuit (*R*3 × 10^−4^) to short circuit (*R*3 × 10^4^), as shown in Figure 5(a). Then, carry out the scanning whose step is −5 k from open circuit (*R*3 × 10^−4^) to short circuit (*R*3 × 10^4^), as shown in Figure 5(b).

According to figures about the relationship between step scanning and FI, for instance, Figure 5, it is composed of two relatively smooth curves and the shortest inflection point appears at about 25 steps (i.e., *R*3 deviates from its own value by about 125 k) no matter the component increases or decreases gradually. However, for prediction on circuits, the situation usually only involves that prediction component deviates its one value by about 50% (i.e., 5 k). In addition to this, the reason why the inflection point appears in the curve is that calculation of the central angle chooses the included angle that is less than 180° when the central angle deviates from itself by 180°. Consequently, appearance of the inflection point does not affect application of the method proposed by this paper to prediction. More importantly, since scanning in Figure 5 chooses the step 5 k, which equals 50% of *R*3 value, and each time index is set as 0.4% of component value generally in prediction, curve in prediction will satisfy features of piecewise linearity well and favorable prediction effect will be obtained eventually.

**Figure 6:**
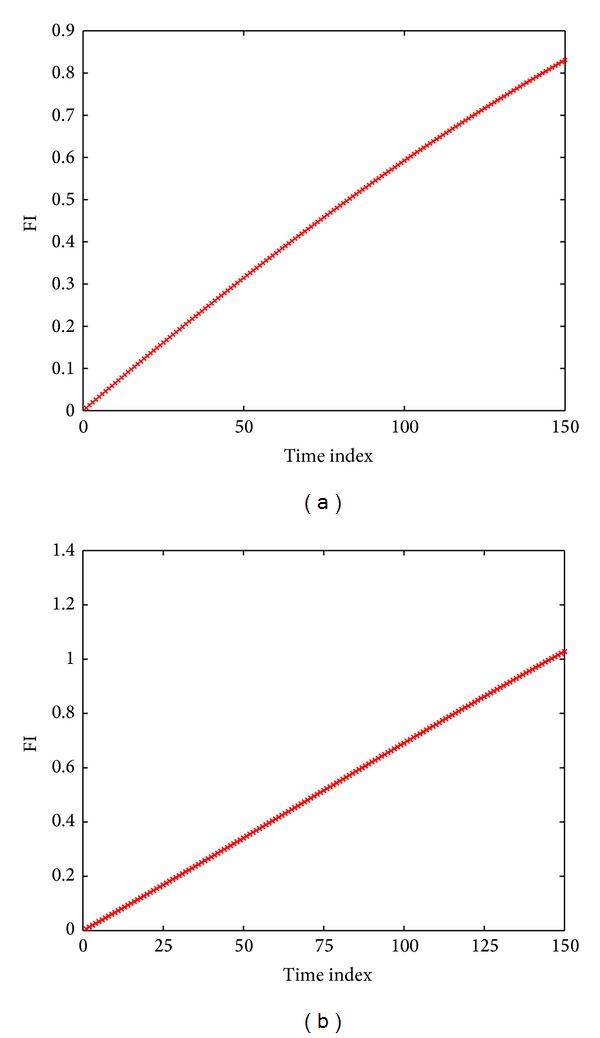
(a) The FI degradation model about the situation that *R*3 deviates from 10 kΩ to 16 kΩ. (b) The FI degradation model about the situation that *R*3 deviates from 10 kΩ to 4 kΩ.

In accordance with the foregoing analyses, calculate FI feature set of *R*3 from the state of no faults and no component displacement to the status that it deviates from its normal value by 60%. Here, each time index is set as 0.4% of component value, as shown in Figure 6. In detail, Figure 6(a) shows that *R*3 deviates from 10 kΩ to 16 kΩ and Figure 6(b) displays that *R*3 deviates from 10 kΩ to 4 kΩ. According to Figure 6, the FI degradation model collected by the round modeling proposed in this paper can be convenient for curve fitting well to do prediction.

### 3.3. FI Collection and Calculation Based on Complex-Field Modeling about the Linear Model

According to analyses of the transfer function, it is found that the round model will degrade into a linear model when the parameter *x*_0_ = 0 in (8), shown as follows:

(10) $$\begin{array}{c} U_{oj}=kU_{or}+U_{\mathrm{int}}\;\;\left(0\leq \sqrt{U_{or}^{2}+U_{oj}^{2}}\leq V_{D}\right), \end{array}$$

where *U*_int⁡_ refers to intercept and *V*_*D*_ is voltage of DC voltage source.

**Figure 7:**
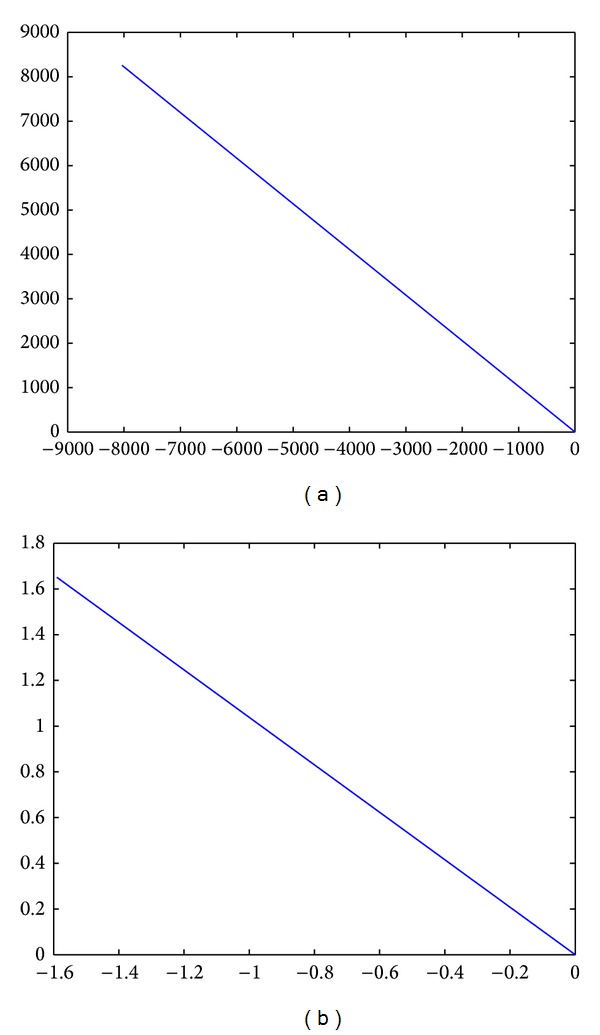
(a) A linear model about parameter scanning voltage of *R*1 from short circuit to open circuit. (b) A linear model about parameter scanning voltage from 5 k to open circuit.

Take the circuit in Figure 4 as an example. When *R*1 is scanned, its linear model is shown in Figure 7.  Figure 7(a) stands for a linear model about parameter scanning voltage from the short circuit *R*3 × 10^−4^ to the open circuit *R*3 × 10^4^, and Figure 7(b) shows a linear model about parameter scanning voltage from 5 k to the open circuit *R*3 × 10^4^.

Based on Figure 7, it is found that slope of the two linear models is basically −1.03. Thus, we know that the component moves along tracks of the linear model which degraded from the round model, when the component degrades.

For judgment about the linear model and the round model, it is impossible to adopt the method of scanning component in prediction either. Thus, this paper puts forward the thought that three points stay in a line if slopes in pairs will be equal which may be used for judgment. Meanwhile, it is supposed that voltage output of the circuit where components are without displacement and faults is set to (*U*_*x*_0__, *U*_*y*_0__), voltage output of short circuit is (*U*_*x*_1__, *U*_*y*_1__) and that of open circuit is (*U*_*x*_2__, *U*_*y*_2__). In doing so, we may obtain the equation of judgment about the situation that the round model degrades into a linear model under an ideal state, shown as follows:

(11) $$\begin{array}{c} \frac{U_{y_{1}}-U_{y_{0}}}{U_{x_{1}}-U_{x_{0}}}=\frac{U_{y_{0}}-U_{y_{2}}}{U_{x_{0}}-U_{x_{2}}}. \end{array}$$

Under an ideal condition, when (11) is satisfied, the component's round model degrades into a linear model. In contrast, when (11) is not satisfied, it is still a round model. However, since tolerance exists in practical situations, (11) is not applicative. Therefore, this paper introduces the following equation to carry out judgment:

(12) $$\begin{array}{c} \frac{U_{y_{1}}-U_{y_{0}}}{U_{x_{1}}-U_{x_{0}}}\in \left(\left(1-\alpha \right)\times \frac{U_{y_{0}}-U_{y_{2}}}{U_{x_{0}}-U_{x_{2}}},\left(1+\alpha \right)\times \frac{U_{y_{0}}-U_{y_{2}}}{U_{x_{0}}-U_{x_{2}}}\right). \end{array}$$

Here, *α* is an empirical constant, which is used to reflect the fluctuation of test data to make theoretical equations satisfy situations of practical tests. Generally, *α* is equal to tolerance value.

When the round model degrades into a linear model, it is impossible to use the central angle as FI any more since the path of component degradation in the complex field has degraded from arc tracks into linear ones. Thus, this paper puts forward that Euclidean distance is used as FI to replace the central angle to carry out prediction when complex field model of the component to be predicted degrades into a linear model, as shown in the following equation:

(13) $$\begin{array}{c} {\text{FI}}_{t}=\sqrt{{\left(x_{t}-x_{0}\right)}^{2}+{\left(y_{t}-y_{0}\right)}^{2}}. \end{array}$$

When a component degrades into *t* time indexes, FI value is set to FI_*t*_, output voltage is *U*_*t*_ = *x*_*t*_ + *jy*_*t*_, and fault-free and displacement-free output is *U*_0_ = *x*_0_ + *jy*_0_.

**Figure 8:**
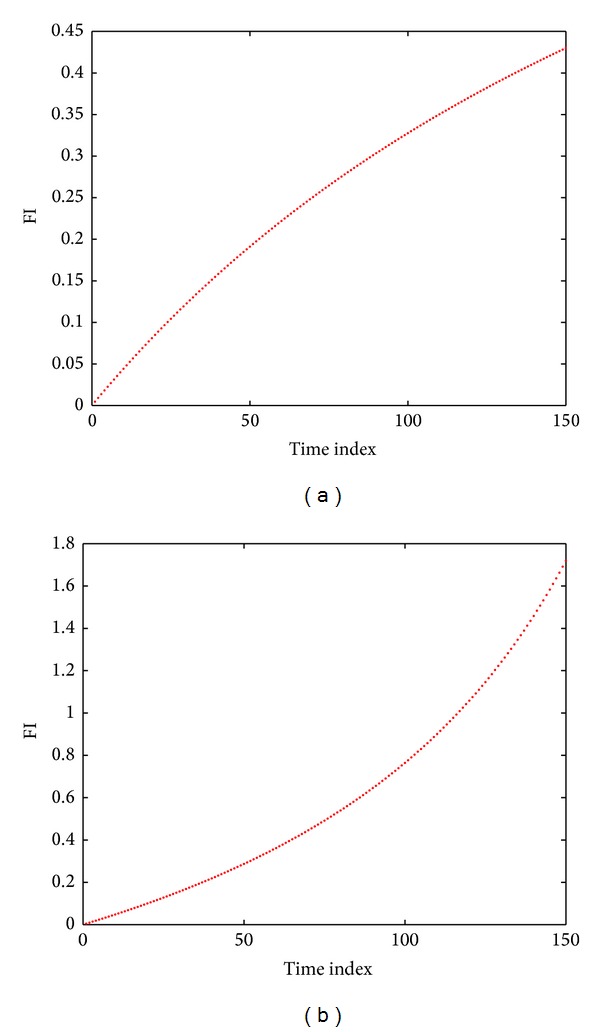
(a) The FI degradation model about the situation that *R*1 deviates from 10 kΩ to 16 kΩ. (b) The FI degradation model about the situation that *R*1 deviates from 10 kΩ to 4 kΩ.

Calculate the FI feature set of *R*1 from the state of no faults and no component displacement to the status that it deviates from its normal value by 60%. Here, each time index is set as 0.4% of component value, as shown in Figure 8. In detail, Figure 8(a) shows that *R*1 deviates from 10 kΩ to 16 kΩ and Figure 8(b) displays that *R*1 deviates from 10 kΩ to 4 kΩ. According to Figure 8, the FI degradation model collected by the linear modeling proposed by this paper can be convenient for curve fitting well to do prediction.

## 4. Degradation Model Adaptation and RUP Prediction Based on Particle Filter

### 4.1. Curve Fitting of the FI Degradation Model of Single Components Analog Circuit

**Figure 9:**
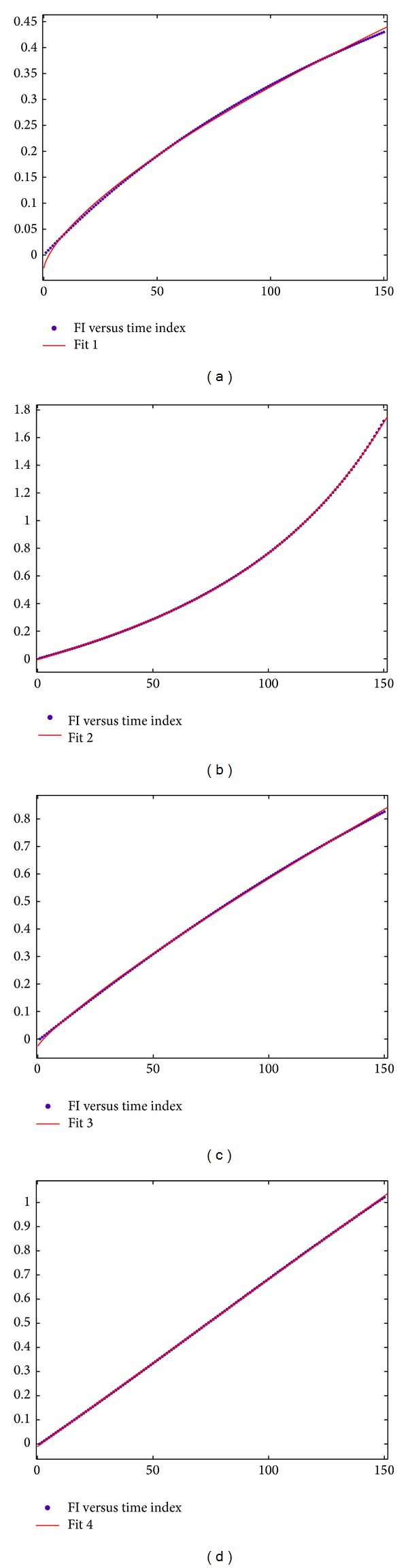
(a) Curve fitting of the FI degradation model about the situation that *R*1 deviates from 10 kΩ to 4 kΩ. (b) Curve fitting of the FI degradation model about the situation that *R*1 deviates from 10 kΩ to 16 kΩ. (c) Curve fitting of the FI degradation model about the situation that *R*3 deviates from 10 kΩ to 4 kΩ. (d) Curve fitting of the FI degradation model about the situation that *R*3 deviates from 10 kΩ to 16 kΩ.

According to analyses in the foregoing chapter and for prediction on single components of analog circuits, the FI degradation model can fit to a convex curve when the component deviates in a forward direction no matter the model is a round one or a degraded linear one under special conditions. When the component deviates in a reverse direction, it can fit to a concave curve. MATLAB is used to carry out curve fitting for these FI data sets. Figures 9(a), 9(b), 9(c), and 9(d) are MATLAB curve fitting of FI set in Figures 8 and 6, respectively. *x*-axis stands for the time index in the process of component degradation and each time index represents 0.4% of original component value. *y*-axis refers to FI value and blue dots are FI set under time indexes of component degradation of 1–150 points, that is, the FI data set obtained in the process in which the component degrades from its initial value to 160% or 40% of its component value. Red line means MATLAB is used for curve fitting of these points. Equations are shown as follows:

(14) $$\begin{array}{c} f\left(x\right)=a\cdot x^{b}+c, \end{array}$$

(15) $$\begin{array}{c} f\left(x\right)=a\cdot \exp\left(b\cdot x\right)+c\cdot \exp\left(d\cdot x\right). \end{array}$$

Fitting formulas and effect of equations related to four different FI data sets are shown in Table 1.

Although fitting effect resulted from use of (15) is better under most ideal conditions, points in FI data sets usually fluctuate since tolerance exits in practical situations. At this time, (15) usually encounters the situation that fitting is impossible. In addition, when data sets approach a line, for instance, the 4th FI set in Table 1, (15) cannot have fitting either. Thus, this paper proposes that fitting of (14) is used for most FI data sets and (15) is used for curve fitting when there is an obvious concave curve, for instance, the 2nd FI set in the foregoing table expressed by Figure 9(b).

### 4.2. Principle of Particle Filter

Although degradation model can be constructed according to FI degradation tendency obtained through curve fitting, FI value has a certain fluctuation due to randomness of test precision, tolerance and Gaussian white noise; moreover, parameters of fitted degradation model curve has subtle changes, which influences precision of prediction. In order to reduce parameter fluctuation in fitted degradation model curve and to improve prediction precision, particle filter is used in this paper to update model.

Principles of particle filter are based on Monte Carlo method. Monte Carlo selected *N* random samples to simulate state space and calculated posterior probability through those *N* samples, which generally included two steps. The first step is prognostics, namely, *N* random samples at time *k* are obtained through state vector at time *k* − 1 according to dynamic model. In other words, *N* random samples at time *k* are obtained through prior probability and weight of *N* samples is calculated at the same time; afterwards, normalization is conducted. The second step is update; namely, resampling is conducted to guarantee that all *N* samples have big weight. In this way, a new sample set can be obtained and be used as posterior sample set at time *k*.

The core of particle filter relies on calculation of importance weight, and calculation formula of importance weight is shown as follows:

(16) $$\begin{array}{c} w_{k}^{i}=\frac{P\left(z_{k}\;|\;x_{k}^{j}\right)P\left(x_{k}^{j}\;|\;x_{k-1}^{i^{*}}\right)}{q\left(x_{k}^{j}\;|\;x_{k-1}^{j^{*}},z_{k}\right)}. \end{array}$$

In (16), *P*(*z*_*k*_∣*x*_*k*_^*j*^) represents similarity, *P*(*x*_*k*_^*j*^∣*x*_*k*−1_^i*^) represents probability density function of transition, and *q*(*x*_*k*_^*j*^∣*x*_*k*−1_^j*^, *z*_*k*_) represents importance density. One optimal importance density will minimize variance of importance weight.

### 4.3. Model Update Based on Particle Filter

In order to reduce parameter fluctuation in fitted degradation model curve and to improve prediction precision, formula of fitted FI degradation tendency curve put forward in this paper is improved as follows:

(17) $$\begin{array}{c} f\left(x\right)=\alpha_{k}\cdot x^{\beta_{k}}+\gamma_{k},\\ \alpha_{k}=\alpha_{k-1}+v_{1},\;v_{1}\in N\left(0,\sigma_{1}\right),\\ \beta_{k}=\beta_{k-1}+v_{2},\;v_{2}\in N\left(0,\sigma_{2}\right),\\ \gamma_{k}=\gamma_{k-1}+v_{3},\;v_{3}\in N\left(0,\sigma_{3}\right), \end{array}$$

(18) $$\begin{array}{c} f\left(x\right)=a_{k}\cdot \exp\left(b_{k}\cdot x\right)+c_{k}\cdot \exp\left(d_{k}\cdot x\right)+e,\\ a_{k}=a_{k-1}+v_{4},\;v_{4}\in N\left(0,\sigma_{4}\right),\\ b_{k}=b_{k-1}+v_{5},\;v_{5}\in N\left(0,\sigma_{5}\right),\\ c_{k}=c_{k-1}+v_{6},\;v_{6}\in N\left(0,\sigma_{7}\right),\\ d_{k}=d_{k-1}+v_{7},\;v_{7}\in N\left(0,\sigma_{7}\right),\\ e_{k}=e_{k-1}+v_{8},\;v_{8}\in N\left(0,\sigma_{8}\right). \end{array}$$

In (17) and (18), *f*(*x*) represents FI at the time index *x*, and *k* stands for the *k*th parameter. Equations (17) and (18) are PF updated formulas of (14) and (15), respectively. According to PF principle, *v* is subject to standard normal distribution and *σ* is covariance value of *v*. PF is used to update model parameters to obtain a new degradation model and realize more accurate prediction on RUP.

## 5. Procedures of the Prediction Method about Single Components Based on Complex Field Modeling in This Paper

**Figure 10:**
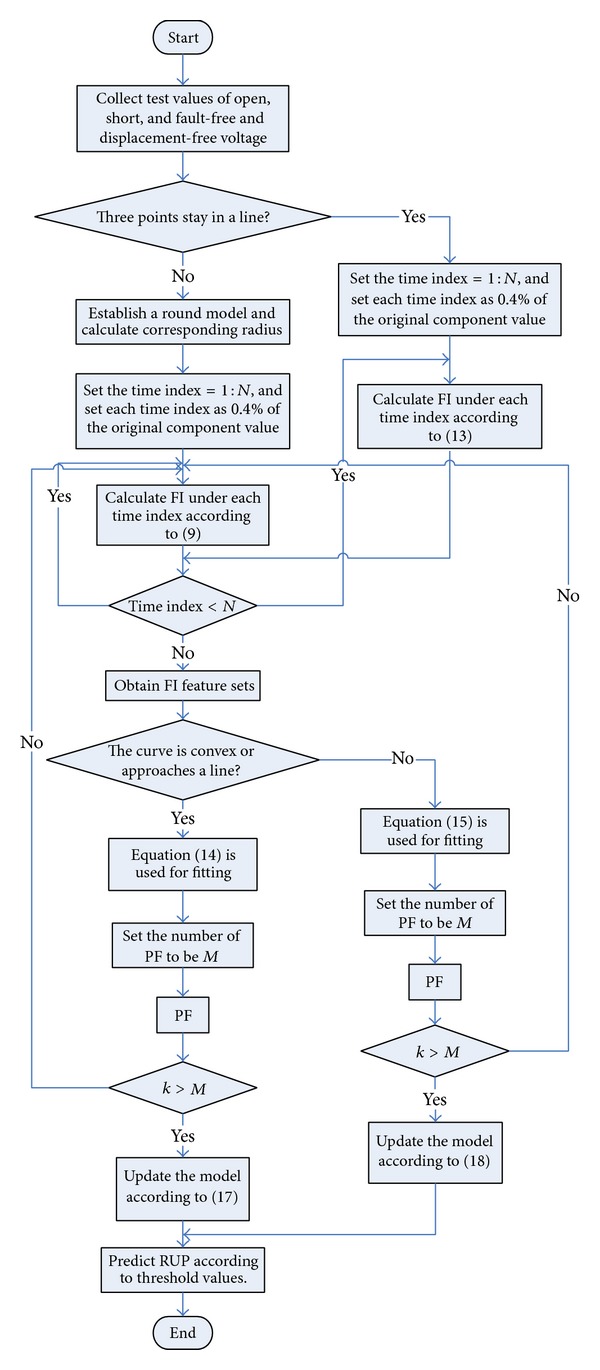
The flow chart of prediction method proposed in this paper about single components based on complex field modeling.

A flow chart about the prediction method about single components based on complex field modeling, which is proposed in this paper, is shown in Figure 10. Its main steps are shown as follows.

**Step 1 (collect output voltage of circuits when the single component to be predicted stays at its basic status).**

Firstly, collect test values of open-circuit voltage, short-circuit voltage, and fault-free and displacement-free voltage of the single components that will be predicted. In simulation, fault-free and displacement-free voltage is the voltage under an ideal condition, and open-circuit voltage and short-circuit voltage are ×10^−4^ and ×10^4^ times higher than predicted component value. In a practical test, voltages at the three conditions are subject to Gaussian distribution since they are affected by tolerance. Thus, according to several tests, average value of each status can be selected as three basic points of complex field modeling in order to improve modeling accuracy in practical tests.

**Step 2 (establish a complex field model).**

According to basic voltage values tested at the three states and (12), judge the complex field model which is a linear one or round one in order to select corresponding ways to calculate FI feature sets.

**Step 3 (collect FI degradation feature sets based on the complex field model).**

When the component that will be predicted satisfies conditions for modeling of the round model, calculate corresponding radius of the round track about voltage motion according to the theory that a round model can be established by three points. Then, use (9) to calculate FI under each time index, and obtain FI feature sets. Here each time index is set to 0.4% of the original component value. When the component that will be predicted satisfies conditions for linear modeling, calculate FI under each time index according to (13), and obtain FI feature values. Here, each time index is set to 0.4% of the original component value as well.

**Step 4 (carry out curve fitting and collect PF parameter sets).**

In accordance with the foregoing FI feature sets, carry out curve fitting. When the component that will be predicted deviates in a forward direction, that is, the curve is convex, (14) is used for fitting. When the curve approaches a line, (14) is still used for fitting. When the component that will be predicted deviates in a negative direction, that is, the curve is concave, (15) is used for fitting. Repeat Steps 3 and 4 to collect a parameter set of PF.

**Step 5 (update the model based on PF).**

According to (17) and (18), update parameters of the obtained parameter sets by using (14) and (15) to obtain optimal curve fitting parameters that are appropriate for prediction.

**Step 6 (predict RUP according to threshold values).**

In accordance with fault threshold values of the component that will be predicted and combine with optimal fitting equation in Step 5. As a result, RUP of the component of analog circuits, which will be predicted, is got.

## 6. Simulation and Experiment

In this chapter, contents mentioned in this paper are demonstrated through simulations and experiments. All simulations are completed by one personal computer with 3 GHz processor and 2 GB memory. Simulation program adopted MATLAB7.1 and OrCAD10.5. In experiments, data under each degradation time index of circuit under test is extracted through Tektronix digital oscilloscope, and data processing is conducted through the mentioned personal computer.

### 6.1. Simulation

**Figure 11:**
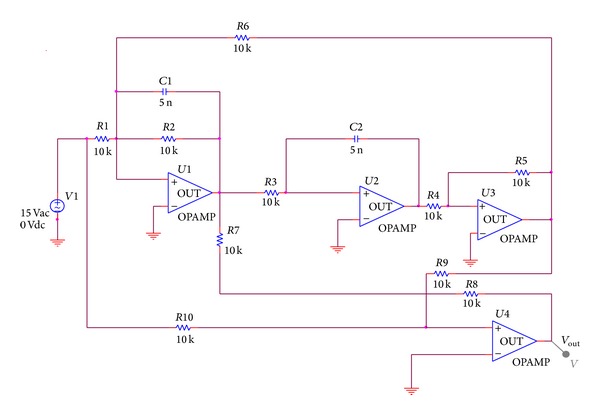
Four-op-amp biquad low-pass filter circuit.

**Figure 12:**
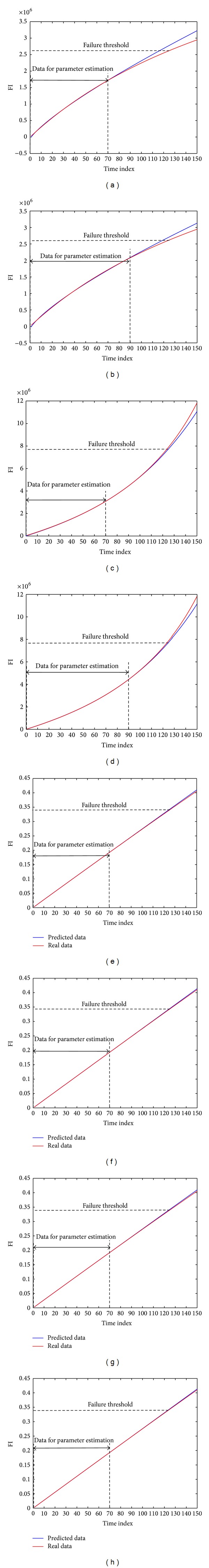
(a) The result obtained when 70 time index data are used to predict 50% of *R*1 deviation in a forward direction. (b) The result obtained when 90 time index data are used to predict 50% of *R*1 deviation in a forward direction. (c) The result obtained when 70 time index data are used to predict 50% of *R*1 deviation in a negative direction. (d) The result obtained when 90 time index data are used to predict 50% of *R*1 deviation in a negative direction. (e) The result obtained when 70 time index data are used to predict 50% of *C*2 deviation in a forward direction. (f) The result obtained when 70 time index data are used to predict 50% of *C*2 deviation in a negative direction. (g) The result obtained when 70 time index data are used to predict 50% of *R*6 deviation in a forward direction. (h) The result obtained when 70 time index data are used to predict 50% of *R*6 deviation in a negative direction.

To verify accuracy of the prediction proposed in this paper under the condition of simulation, select Figure 11 as the circuit to be predicted. In detail, basic setting of components is shown in Figure 11 and all components stay in the tolerance range [−10%, 10%]. Firstly, it is considered that the single component *R*1 is a degraded one and it is set that each time index of *R*1 deviates from its own component value by 0.4%. In accordance with (12), it may be judged that the FI degradation model of *R*1 cannot establish a round model but a linear one. Therefore, (13) is used to establish the FI degradation model. When the component *R*1 deviates from their initial component values in a forward direction, we know that prediction curves are shown as Figures 12(a) and 12(b) according to (17), and when the component *R*1 deviates from their original component values in a negative direction, we know that prediction curves are shown as Figures 12(c) and 12(d) according to (18). Then, it is considered that *C*2 is a degraded component and it is set that each time index of *C*2 deviates from its own component value by 0.4%. According to (12), it may be judged that the FI degradation model of *C*2 may establish a round model. Thus, the FI degradation model of *C*2 is established by using (9). Based on (17), we know the prediction curve is shown as Figure 12(e) when the component *C*2 deviates from their initial component values in a forward direction. Similarly, the prediction curve is shown as Figure 12(f) when the component *C*2 deviates from their initial component values in a negative direction in accordance with (17). Next, it is considered that *R*6 is a degraded component and it is set that each time index of *R*6 deviates from its own component value by 0.4%. In accordance with (12), it may be judged that the FI degradation model of *R*6 can establish a round model. Thus, the FI degradation model is established by using (9). On the basis of (17), we know the prediction curve is shown as Figure 12(g) when the component *R*6 deviates from their initial component values in a forward direction. Similarly, the prediction curve is shown as Figure 12(h) when the components *R*6 deviates from their initial component values in a negative direction in accordance with (17).

Table 2 shows effect of prediction on each curve in Figure 12. According to Table 2, it is shown that effect of prediction will be better when data used for prediction increase. For instance, when the component *R*1 that will be predicted deviates from 10 kΩ to 15 kΩ in a forward direction or to 5 kΩ in a negative direction; that is, *R*1 deviates from its own value by 125 time indexes, and the number of data used for prediction is 70, the effect of prediction on RUP is better than that when 90 time indexes are used. In addition, when a round model is used, effect of prediction is better compared to the effect when a linear model is used. For example, when the component *C*2 that will be predicted deviates from 5 nF to 7.5 nF in a forward direction or to 2.5 nF in a negative direction, that is, *C*2 deviates from its own value by 125 time indexes, the effect of prediction on RUP is better than that of the prediction on *R*1 under the linear model. When the component *R*6 that will be predicted deviates from 10 kΩ to 15 kΩ in a forward direction or to 5 kΩ in a negative direction, that is, *R*1 deviates from its own value by 125 time indexes, the effect of prediction on RUP is better than that of the prediction on *R*1 under the linear model. FI calculation of the prediction method in [10] only adds weights of Euclidean distance between features of components' deviation status and features of ideal conditions, which is similar to the linear model method proposed in this paper. Therefore, in accordance with analyses of circuit structure, it is found that situations satisfying the round model are in the majority, while there is only one situation satisfying the linear model generally for prediction on single components. Thus, effect of the prediction method proposed by this paper is better than the one in [10] for analog circuit prediction of single components.

### 6.2. Experiment

**Figure 13:**
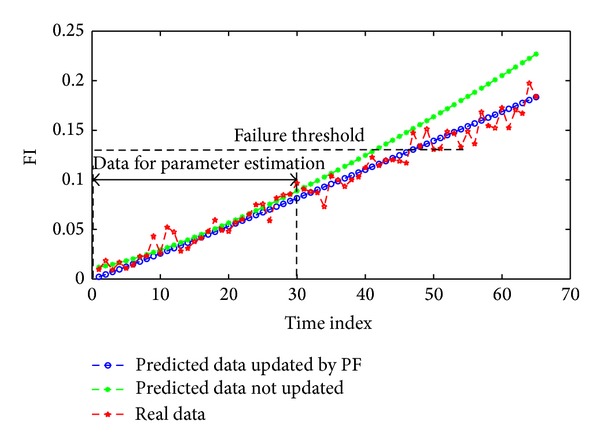
The result obtained when 30 practically tested data about time indexes are used to predict 20% of *R*3 deviation in a forward direction.

To verify effect of the prediction method proposed by this paper when it faces practical data, practical analog circuits are selected as experimental objects. Setting of circuit components is shown in Figure 11. In the experiment, Tektronix digital oscilloscope is used to collect output voltage waveform. Sampling rate of the oscilloscope is 1 GS/s and bandwidth is 100 M. Since most prediction models about single components of analog circuits are round models, practical tests on round models are taken for example here. It is considered that the resistance *R*6 is a degraded component and it is increased by 40 Ω each time to make itself change from 10 kΩ to 12 kΩ. Collecting data where the component is changed from 10 kΩ to 11.2 kΩ as prediction data, predict the prediction curve when *R*6 changes into 12 kΩ, and compare the prediction curve with the practically collected data from 10 kΩ to 12 kΩ, as shown in Figure 13.

In practical tests, parameter calculation of the round model is often obtained by averaging after several tests. In the foregoing experiments for establishment of the round model about *R*6, the practical radium calculated after averaging via several practical tests only deviates from the radius obtained by ideal simulation by about 0.36%. Thus, it will not affect FI calculation of the round model basically. As shown in Figure 13, the blue curve represents the prediction curve passing PF parameter update, the green curve stands for the prediction curve that does not pass PF parameter update and the red curve denotes the curve about the FI degradation model established for a practical test. Based on Figure 13, since tolerance exits, FI point calculated by voltage values in the test fluctuates to some extent compared with the simulated point. Thus, although PF parameter update does not have obvious effect in simulation, the curve passing PF parameter update has obvious deviation compared to the fitting curve of the FI degradation model established in a practical test. Besides, it is obvious that the curve passing PF parameter update can carry out more accurate prediction on the FI degradation model. Compared with practical test effect in [10], the FI degradation model established based on the round model has more stable and more accurate prediction effect in practical tests since the model is featured by better piecewise linearity.

## 7. Conclusion

This paper proposes a prediction method about single components of analog circuits based on complex field modeling. Aiming at the feature that faults of single components hold the largest number in analog circuits, the method regards continuous degradation process of each component as sum of discrete degradation process approximately. Then, it starts with circuit structure, analyzes the transfer function of circuits, and implements complex field modeling. Next, by an established parameter scanning model related to complex field, it analyzes the relationship between parameter variation and degeneration of single components in the model in order to obtain a more reasonable FI feature set via calculation. According to the newly obtained FI feature set, it establishes a novel model about degeneration trend of analog circuits' single components. At last, it uses particle filter to update parameters for the model and predicts RUP of analog circuits' single components. The difference between the perdition method proposed in this paper and previous ones is that previous methods only extract some features from predicted circuit output and obtain feature FI. Instead, the method in this paper carries out complex field modeling by analyzing transfer function of circuits and analyzes the relationship between parameter variation and degeneration of single components via parameter scanning for the complex field model. The FI feature sets obtained in this way are fully related to circuit structure, which makes calculation about FI more reasonable. Hence, accuracy of the whole prediction is improved. Finally, this paper verifies the foregoing content by simulation and experiment.

## Figures and Tables

**Table 1:** Fitting formulas and effect of equations related to FI data sets.

FI	Degradation components	General formula	Coefficients (with 95% confidence bounds)	RMSE
1	*R*1 (10 kΩ to 16 kΩ)	(14)	*a* = 0.01484 (0.01388, 0.0158) *b* = 0.6866 (0.6748, 0.6984) *c* = −0.02625 (−0.02997, −0.02253)	0.003632

2	*R*1 (10 kΩ to 4 kΩ)	(15)	*a* = 0.1746 (0.1721, 0.177) *b* = 0.01529 (0.0152, 0.01538) *c* = −0.1806 (−0.1827, −0.1785) *d* = −0.01446 (−0.01528, −0.01364)	0.002864

3	*R*3 (10 kΩ to 16 kΩ)	(14)	*a* = 0.01192 (0.01145, 0.01239) *b* = 0.8539 (0.8465, 0.8613) *c* = −0.01988 (−0.02303, −0.01674)	0.004004

4	*R*3 (10 kΩ to 4 kΩ)	(14)	*a* = 0.007 (0.006887, 0.007113) *b* = 0.9976 (0.9945, 1.001) *c* = −0.00408 (−0.005341, −0.002819)	00.001902

**Table 2:** Effect of simulation and prediction on single components.

Figure 12	Degradation components	kind of modeling	General formula	Data for parameter estimator (time index)	Actual RUP	RUP prediction	RUP prediction error
a	*R*1 (10 kΩ to 15 kΩ)	Line	(17)	70	125	116	9
b	*R*1 (10 kΩ to 15 kΩ)	Line	(17)	90	125	120	5
c	*R*1 (10 kΩ to 5 kΩ)	Line	(18)	70	125	128	3
d	*R*1 (10 kΩ to 5 kΩ)	Line	(18)	90	125	127	02
e	*C*2 (5 n to 7.5 n)	Circularity	(17)	70	125	125	00
f	*C*2 (5 to 2.5 n)	Circularity	(17)	70	125	125	00
g	*R*6 (10 k to 15 k)	Circularity	(17)	70	125	125	00
h	*R*6 (10 k to 5 k)	Circularity	(17)	70	125	125	00
